# New computational approaches to understanding molecular protein function

**DOI:** 10.1371/journal.pcbi.1005756

**Published:** 2018-04-05

**Authors:** Jacquelyn S. Fetrow, Patricia C. Babbitt

**Affiliations:** 1 Office of the President, Albright College, Reading, Pennsylvania, United States of America; 2 Department of Bioengineering and Therapeutic Sciences, University of California San Francisco, San Francisco, California, United States of America

## Defining function

Function is like beauty—its definition lies in the eye of the beholder or, in this case, the researcher. At the broadest level, we define organismal function—the function that the protein plays in the overall organism. This function can be observed by understanding the impact on the organism of deletion or mutation of the protein. Physiological function is the function the protein plays in pathways, such as metabolic or signaling pathways. Another level of function is the cellular level. Approaches to understanding cellular function attempt to identify a protein’s interaction partners and its location within the cell. Biochemical or molecular function is another level of function, one that identifies the molecular functional details of a functional site, including reaction mechanism, substrate binding, and molecular details of the binding of regulatory molecules. The International Union of Pure and Applied Chemistry (IUPAC) Enzyme Classification (EC) system was an early approach to identifying molecular function. The Gene Ontology (GO) system of classifying function recognizes ways of defining function, using distinct cellular components, molecular function, and biological process hierarchies [1]. The emphasis in the collection of research articles that comprise this Focus Feature is on understanding the different molecular functions that can exist within a protein superfamily and the features that define those differences.

## Brief history of protein function identification/prediction

With the ongoing genome sequencing projects, the number of predicted proteins continues to increase exponentially. Understanding the biological role of these protein sequences requires an understanding of their functions, a goal that has resulted in many experimental and computational methods to evaluate gene product function. Large-scale experiments have aided in identification of protein-protein interactions, nonprotein binding partners, and expression levels, each of which define some manner of cellular function of the gene product. Other large-scale projects, such as the Enzyme Function Initiative (EFI, [2]) have focused on molecular or biochemical function.

Computational methods also abound. Our goal here is not to provide a thorough review but rather to provide a brief overview of new work in the field, with illustrative examples. The most commonly used approach is function annotation transfer, exemplified by common application of BLAST and Position-Specific Iterative BLAST (PSI-BLAST) [3,4]. In its simplest form, pairwise sequence similarity between 2 proteins is determined and, if the proteins are similar enough (i.e., if the similarity between them is deemed significant), function annotation is transferred from one protein to the other. There are many other more sophisticated approaches that can be applied for annotation transfer (e.g., ClustalOmega [5]) that take advantage of multiple alignments or alignment of a query protein to a probabilistic profile, as well as a plethora of other approaches, all of which are useful in various ways [6]. On the other hand, inappropriate application of annotation transfer has resulted in substantial annotation errors because the annotation transferred is not substantiated by available evidence [7]. Most often, misannotations arise from transfer of a more detailed molecular function than is warranted.

Other approaches to understanding molecular function involve motifs, either sequence motifs exemplified by PRINTS [8] and PROSITE [9] or structural motifs exemplified by Fuzzy Functional Forms [10] and Patterns in Non-homologous Tertiary Structures (PINTS) [11], or methods that use structure to inform sequence, such as Catalytic Site Atlas [12] and Enzyme Function Inference by a Combined Approach (EFICAz) [13]. All of these methods identify patterns of amino acids that correlate with a given function. To predict a functional site, a sequence or structure of unknown function is evaluated for how well it matches each motif. Many methods for evaluation of the match between motif and sequence (or structure) have been developed.

Finally, several groups have developed methods to cluster proteins into functionally relevant groups. These approaches—exemplified by Genome Modelling and Model Annotation (GEMMA) [14], Subfamily Classification in Phylogenomics (SCI-PHY) [15], Active Sites Modeling and Clustering (ASMC), [16], and Two-Level Iterative Clustering Process (TuLIP) [17]—cluster proteins using sequence, structure, evolutionary relationships, genomic context, or other metrics and correlate those clusters with some level of protein function. The goal of these approaches is not to predict function per se but rather to cluster proteins into functionally relevant groups, often using network methods [18,19], thus informing function annotation from an assumption that detailed molecular functional information can be transferred between members of the cluster. This approach has thus far most often applied to the molecular function of enzyme superfamilies.

## Why molecular function?

The emphasis of this Focus Feature is molecular function, particularly for enzymes. A primary goal of molecular function analysis is to identify and understand the role of mechanistic or functional determinants, patterns of amino acids that distinguish one functional group from another. These are typically the amino acids that enable substrate binding and catalytic mechanism. Such features distinguish one molecular functional family from another. Their identification has long been the subject of expert analysis in the pharmaceutical and structure-based drug discovery programs, as these features are used to design the most specific inhibitors for a given functional site.

Understanding molecular functional determinants would also provide a better understanding of evolution. Contemporary protein superfamilies are the result of numerous genetic events, including gene duplications and horizontal transfers. The relationship between molecular function and evolution has long been debated [20]. Indeed, an underlying assumption in many annotation transfer approaches such as those mentioned above is that molecular function can be transferred from one family member to another. As has been clearly demonstrated [7,21–23], this assumption does not always hold, especially at detailed levels of molecular function. The ability to computationally identify molecular function at the most detailed level would provide the opportunity to compare trees and branches of evolutionary pathways, as has been illustrated by the analysis of the carbohydrate kinases [24].

## Hierarchy in molecular function

Hierarchy is inherent in molecular function. Early on, this hierarchy was recognized in the IUPAC EC system, in which each enzyme was assigned a 4-digit EC number, W.X.Y.Z. In the EC system, wherein W represents 1 of 6 main types of chemical reactions (classes) to which the enzyme belongs, X indicates a more detailed level of reaction, i.e., subclass, Y indicates the sub-subclass (typically reaction specificity), and Z is the serial number of the enzyme in its sub-subclass and describes substrate specificity, including pertinent cofactors.

Hierarchy is also represented in the GO system of molecular function classification [1]. As an example from the paper in this Focus Feature series by the Orengo group [25], the GO molecular function ontology term GO:0008800 represents “beta-lactamase activity.” This function is further subdivided into GO:0033250 “penicillinase activity” and GO:0033251 “cephalosporinase activity,” representing a hierarchy of molecular function in this superfamily from less specific to more specific.

**Fig 1:**
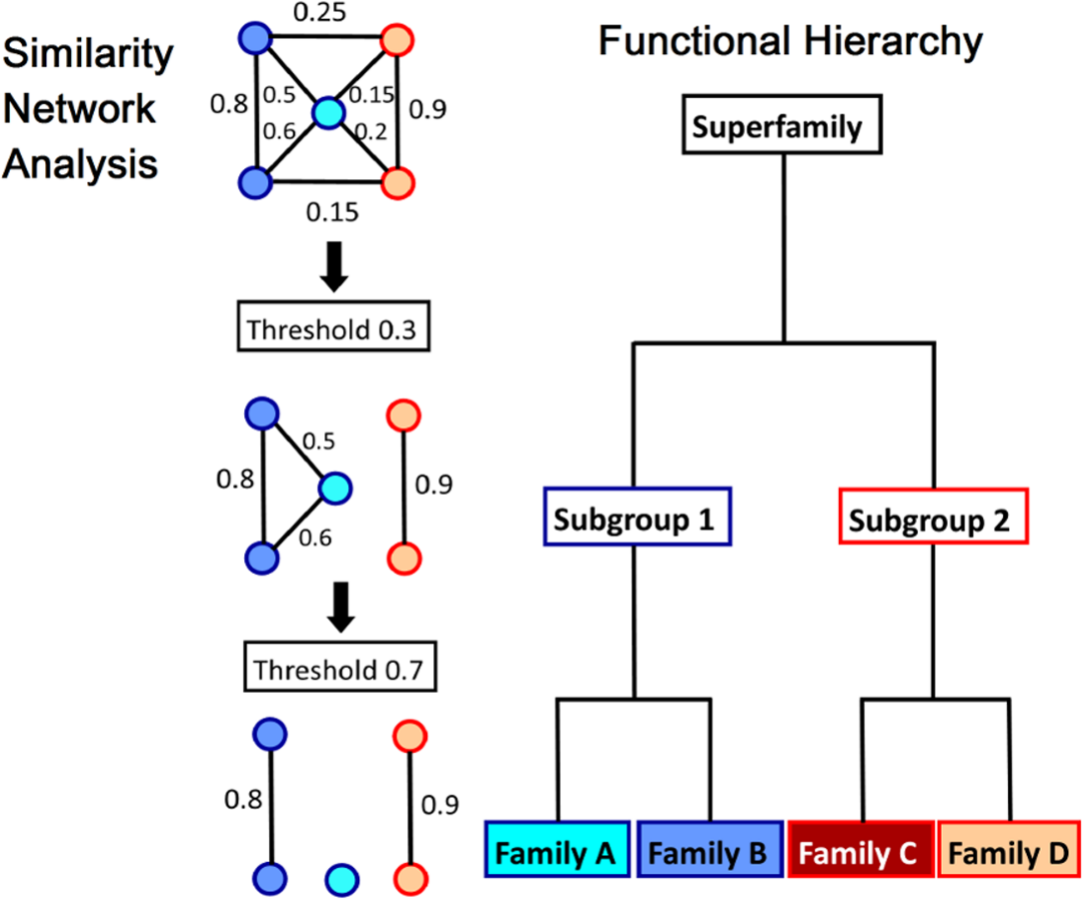
An illustration of the concept of molecular functional hierarchy and its correlation with network analysis. Similarity network analysis (left) uses edge thresholds to identify clusters that are progressively more similar to each other. As an example of molecular functional hierarchy, the Structure-Function Linkage Database (SFLD) hierarchy is shown on the right. Ideally, network clustering would capture the biologically relevant functional boundaries that would correlate with defined level of functional hierarchy, such as those defined by SFLD or the Gene Ontology (GO) hierarchies. (Note: the figure is illustrative and is not meant to suggest that an edge threshold of 0.3 correlates with the subgroup level of the SFLD hierarchy. One challenge in this field, illustrated by some of the papers in this Focus Feature, is which edge metric and threshold correlate with which levels of functional hierarchy).

The Structure-Function Linkage Database (SFLD) also defines a molecular functional hierarchy, in this case as superfamily, subgroup, and family (Fig 1) [26]. Superfamilies represent broad groups of proteins, such as the enolases or glutathione transferases, which are homologous (descended from a common ancestor) and which share a common reaction step or other chemical capability. Members of an SFLD subgroup share some, but not necessarily all, reaction steps, while family members share all or almost all steps of a reaction mechanism and perform the same molecular function. Some superfamilies within the SFLD database have been utilized as a “gold standard” for validation of approaches to predicting molecular function [7,14,27].

A key feature of annotation transfer methods is that the level of functional hierarchy must correlate with the similarity method and mathematical and statistical comparison that is being done [28]. An illustrative comparison between similarity network analysis [19] and the SFLD hierarchy is shown in Fig 1. In the original ASMC publication, by de Melo-Minardi and coworkers (contributors to this Focus Feature), for instance, the level of the functional hierarchy is called out explicitly for each protein superfamily studied [16]. For Multi-level Iterative Sequence Searching Technique (MISST), the topic of one of the papers in this Focus Feature series [29], the aim is to identify functional families using SFLD curated families as the gold standard definition of proteins that share all or almost all catalytic reaction steps. Defining the level of molecular function (Fig 1) being targeted by a given method is essential to interpretation of the results.

## Focus feature on molecular function prediction

The goal of this Focus Feature is to present some recent work on determining molecular function and the details of features that distinguish one molecular function from another in a superfamily. Three research groups take the approach of clustering proteins into functionally relevant groups. The fourth research group describes a computational approach to enumerating potential reaction products in a protein superfamily, i.e., an approach to identifying what each of those functionally relevant clusters within a superfamily might do.

The contribution by Orengo and her colleagues describes their most recent work on clustering the beta lactamase protein superfamily [25]. Building on their previously published FunHMMer [30] work, this analysis distinguishes the A, C, and D classes of serine beta lactamases. Filtering these sequences using CD-HIT [31] at a 60% identity threshold divides Class A into 151 clusters. Nine of these correlate with 15 known types of the Class A beta lactamases. One hundred and forty-two are newly identified. Orengo and her colleagues then tackle the difficult problem of identification of the residues that confer antibiotic resistance and differentiate these clusters. As described in the Focus Feature contribution, an active site structure profile (ASSP) is used to identify key functional positions near the active site, and a second shell parsimony approach (SSPA) to identify additional mutations and drive more detailed functionally relevant clustering—to identify types and variants within each class. Such approaches are broadly useful for identifying mechanistic or functional determinants (such as those that confer antibiotic resistance).

The contribution of de Melo-Minardi and her colleagues [32] describes the development of a new method for functionally relevant clustering that evolved from their published ASMC approach [16] to identifying functionally relevant clusters in the PFAM protein families database [33] superfamilies. Comparative models are built for each member of a PFAM superfamily that is at least 30% sequence identical to another superfamily member of known structure. Genetic programming (GP) is used to explore the optimal combinations of evidence regarding function (sequence, structural, and genomic context and active site information) that give the best separation of clusters. Spectral clustering divides the family into a specified number of clusters; mutual information identifies the optimal number of clusters. This method, called a GP (for genetic programming) approach by the authors, is applied to a variety of superfamilies, including nucleotidyl cyclases, kinases, serine proteases, enolases, and crotonases, with good success.

A third method, MISST [29], for clustering proteins in functionally relevant ways is also part of this Focus Feature series. This method implements an approach called Deacon Active Site Profiler (DASP), [34]. DASP utilizes the concept of active site profiling [35] to identify sequences that share functional site features in common. This method was built on a key observation that isofunctional clusters, groups of sequences that share most details at their molecular sites, self-identify in DASP searches [17]. This observation is built into an iterative search process, MISST, that both aggregates sequences and identifies when a cluster should be subdivided because it is composed of more than one isofunctional group. In this contribution, the approach is applied to the peroxiredoxin superfamily. Notably, this approach does not start with all members of a superfamily, as do the first 2 methods, but rather aggregates sequences from the GenBank database into functionally relevant clusters within a superfamily.

Notably, the above methods are not function prediction methods because, once functionally relevant clusters are identified, the key question remains to be answered: What do the proteins in each cluster actually do? That is, what is the reaction mechanism of the proteins in each cluster, and what are the substrates utilized and products produced by each cluster? Jacobson and his colleagues present an approach to answering that question, which builds on previous work on the triterpenoid synthases that utilized docking to identify potential substrates and functional families [36]. Their contribution to this Focus Feature series describes the use of structural modeling, virtual reactions, and energy calculations to systematically enumerate plausible terpenoid carbocations, thus exploring chemical space of the products of the monoterpenoid synthases [37]. Five of the 74 identified skeletons are found in natural products. Others can be connected to substrates by small carbocation rearrangements already known to occur. The authors hypothesize that these skeletons may represent currently unidentified natural products of one or more of the terpene synthases for which detailed molecular function is uncharacterized. The enumeration algorithm they describe, iGEN, could be used to create carbocations in the active site of an enzyme, which may allow both the prediction of novel terpenoid skeletons and matching them to a terpenoid synthase of unknown function. Indeed, a proof of concept of this approach has been published [38].

## Commonalities in approach: Functional site signatures

**Fig 2:**
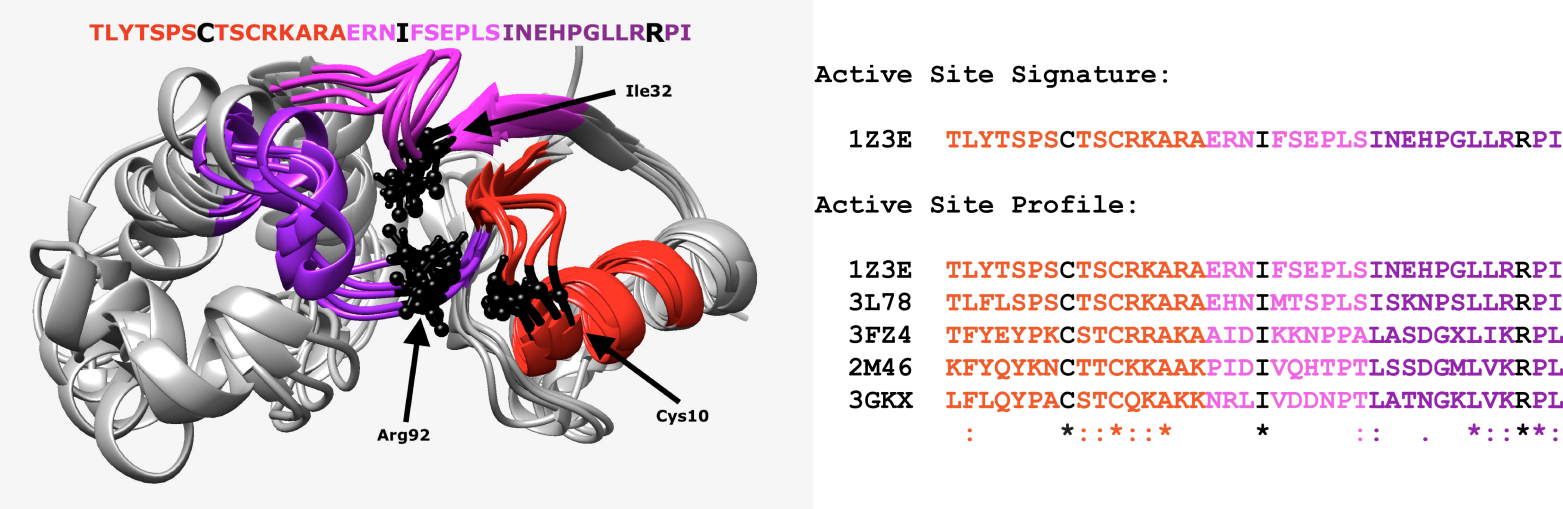
Specificity determining or “signature” positions are amino acids directly involved in an enzyme’s activity. Three such residues are shown as black side chains (left) for 5 arsenate reductase enzymes. Residues within close structural space are shown as colored fragments. The sequences of those colored fragments create the active site signature (right), a residue sequence originally defined by Cammer and colleagues [35]. Signatures can be aligned to create a profile, which allows direct comparison of residues in and near the active site. This active site profile concept is used by 3 of the approaches for functionally relevant clustering of protein superfamilies included in this Focus Feature. (The authors gratefully acknowledge Mikaela Rosen for creating these figures).

A common feature of several of the clustering approaches in this Focus Feature is the definition of a “functional site signature.” At its simplest, a signature consists of the “mechanistic determinants” or “specificity-determining positions” or “functional determinants”—only those residues that are actively involved in substrate binding or catalytic mechanism (as illustrated for an arsenate reductase, Fig 2). Godzik and colleagues called these residues “signature positions” in their work on the carbohydrate kinases [24]. A more encompassing definition of an active site signature was defined by Cammer and colleagues [35] and includes all residues within 10 Å of the centers of geometry of 3 key specificity-determining residues. Similarly, Orengo’s ASSP algorithm identifies all residues within 8 Å of the key active site serine in the beta lactamases to build the ASSP.

Each of the 3 clustering approaches use the concept of active site profiling, including a multiple sequence alignment that represents the active site composition for each protein in the family. This alignment is termed an active site profile (ASP) by Fetrow and colleagues [35] and an ASSP by Orengo and colleagues [25]. In the Orengo method, ASSP is used to identify functionally important residues. In de Melo-Minardi’s contribution, these residues are used to optimize the separation of clusters [32]. In MISST, the profiles are used by DASP to search the sequence databases to identify protein sequences that contain fragments that are statistically similar to those in the query profile [29]. No matter how such profiles are used, the definition of such functionally relevant signatures is valuable in informing our understanding of the mechanistic differences between functionally relevant clusters.

## Challenges and a view to the future

A key challenge for clustering proteins in functionally relevant ways is the identification of the number of clusters that are optimal for defining all distinct molecular functions within a protein superfamily—that is, the number of clusters that captures biologically relevant functional boundaries that correlate with the level of molecular functional hierarchy being pursued (Fig 1). The previously published ASMC method utilized the full hierarchy of clusters, with manual pruning of branches [16], while de Melo-Minardi’s current contribution solves this problem using mutual information to identify an appropriate number of clusters [32]. FunHMMer identified many families in the beta lactamases, and Orengo and her colleagues used sequence identity and active site residue analysis to distinguish and/or combine these into functionally relevant clusters in the hierarchy. MISST is an aggregative, rather than divisive, clustering approach, starting with a small, representative set of proteins, aggregating new members, and identifying the point at which a cluster contains more than one isofunctional cluster. Thus, it only has to identify when a cluster is “complete,” rather than at what level the clustering correlates with molecular function.

Another key challenge for methods aimed at molecular function identification is validation. Isofunctional clusters, defined as a family by SFLD, have been defined and experimentally validated for only a small number of families. For example, the enolase superfamily and its functionally relevant families have been studied extensively [39–41], and these functionally relevant groups (along with those of other SFLD superfamilies) have been used for validation as well [27]. More recently, the peroxiredoxins [42], FGGY carbohydrate kinases [24], and matrix metalloproteins [43] have also been described at a detailed molecular functional level and could serve as additional validation standards.

Clearly, our understanding of molecular function continues to evolve. Each of the methods described in this Focus Feature builds on previous work, and each continues to aid our understanding of molecular function and relationships between functionally relevant clusters within a protein superfamily. For the future, much new work seeks to improve automated and “agnostic” approaches for functional inference, while other communities, such as Critical Assessment of Function Annotation (CAFA) [6], have developed improved approaches for evaluation of such methods. And while the “messiness” of biology continues to challenge general solutions for identifying functional boundaries, new approaches such as those described in the Focus Feature offer new directions for exploration. The need for continued progress remains clear, however, as the volume of sequencing will continue to increasingly outpace experimental validation.

**The Protein Molecular Function Prediction Focus Feature consists of the following 4 research articles:**

Novel computational protocols for functionally classifying and characterising serine beta-lactamases. David Lee, Sayoni Das, Natalie L. Dawson, Dragana Dobrijevic, John Ward, Christine Orengo. Published 22 Jun 2016. *PLOS Computational Biology*. http://dx.doi.org/10.1371/journal.pcbi.1004926

Isofunctional protein subfamily detection using data integration and spectral clustering. Elisa Boari de Lima, Wagner Meira Júnior, Raquel Cardoso de De Melo-Minardi. Published 27 Jun 2016. *PLOS Computational Biology*. http://dx.doi.org/10.1371/journal.pcbi.1005001

Defining the product chemical space of monoterpenoid synthases. Boxue Tian, C. Dale Poulter, Matthew P. Jacobson. Published 12 Aug 2016. *PLOS Computational Biology*. http://dx.doi.org/10.1371/journal.pcbi.1005053

An atlas of peroxiredoxins created using an active site profile-based approach to functionally relevant clustering of proteins. Angela F. Harper, Janelle B. Leuthaeuser, Patricia C. Babbitt, John H. Morris, Thomas E. Ferrin, Leslie B. Poole, Jacquelyn S. Fetrow. Published 10 Feb 2017. *PLOS Computational Biology*. http://dx.doi.org/10.1371/journal.pcbi.1005284
